# Prehabilitative versus rehabilitative exercise in prostate cancer patients undergoing prostatectomy

**DOI:** 10.1007/s00432-023-05409-3

**Published:** 2023-09-15

**Authors:** Favil Singh, Robert U. Newton, Dennis R. Taaffe, Pedro Lopez, Jeff Thavaseelan, Matthew Brown, Elayne Ooi, Kazunori Nosaka, Dickon Hayne, Daniel A. Galvão

**Affiliations:** 1 School of Medical and Health Sciences, Edith Cowan University, 270 Joondalup Drive, Joondalup, WA 6027 Australia; 2 Exercise Medicine Research Institute, Edith Cowan University, Joondalup, WA Australia; 3 School of Human Movement and Nutrition Sciences, University of Queensland, St Lucia, QLD Australia; 4 Pleural Medicine Unit, Institute for Respiratory Health, Perth, WA Australia; 5 Perth Urology Clinic, Perth, WA Australia; 6 Fiona Stanley Hospital, Murdoch, WA Australia; 7 UWA Medical School, University of Western Australia, Crawley, WA Australia; 8 Swan Urology, Perth, WA Australia

**Keywords:** Prostate cancer, Exercise, Surgery, Prehabilitation, Prostatectomy, Incontinence

## Abstract

**Purpose:**

The study compared the efficacy of commencing supervised exercise in men with prostate cancer before and after prostatectomy on objective and patient-reported outcomes, hospital length of stay, and urinary incontinence.

**Methods:**

Forty-one men were randomised to a 6-week prehabilitation or rehabilitation exercise programme. Prehabilitation involved resistance and aerobic exercise thrice weekly pre-surgery, while rehabilitation comprised the same commencing 6-weeks post-surgery. Assessments included strength, function (chair rise, stair climb, 400-m, 6-m usual, fast, and backwards walk), body composition, fatigue and quality of life, undertaken at pre-surgery, early post-surgery and late post-surgery phase, with urinary incontinence (24-h pad test) assessed at 2, 6, and 12-weeks post-surgery. Intention-to-treat and sensitivity analyses were undertaken.

**Results:**

Of thirty-eight men (48–73 years), 29 completed all assessments with most undergoing robotic-assisted laparoscopic prostatectomy (92.1%). In the pre-surgery phase, prehabilitation improved muscle strength (leg press: 17.2 kg; chest press: 2.9 kg; p ≤ 0.001), 400-m, chair rise, 6-m fast and backward walk tests (p ≤ 0.001–0.028). Strength and function declines in the early post-surgery phase were maintained late post-surgery. Rehabilitation showed declines of these outcomes after surgery with improvement late post-surgery (leg press: 14.6 kg, p < 0.001; chest press: 6.8 kg, p < 0.001; 400-m walk: -12.0 s, p = 0.005), resulting in no difference between groups at 12 weeks. There were no significant differences between groups for patient-reported outcomes, hospital length of stay or urinary incontinence.

**Conclusion:**

Pre-surgical exercise enhanced strength and function, protecting against post-surgery declines. Although exercise post-surgery is beneficial for recouping strength and function, where possible men undergoing prostatectomy are encouraged to exercise pre-surgery.

**Trial registration:**

ACTRN12617001115325 registered 31 July 2017.

**Supplementary Information:**

The online version contains supplementary material available at 10.1007/s00432-023-05409-3.

## Introduction

Prostate cancer is the most diagnosed cancer in men in Western countries such as the United States, United Kingdom and Australia (AIHW 2022; Siegel et al. 2022). Prostatectomy, the main treatment for localised disease, results in various adverse effects such as incontinence, sexual dysfunction, and reduced physical function which impacts quality of life (QoL) (Gacci et al. 2009, 2023). Exercise can improve physical function, QoL and alleviate adverse effects such as fatigue as a result of prostatectomy (Singh et al. 2017b; Santa Mina et al. 2018), androgen deprivation therapy (Galvão et al. 2010) and radiation therapy (Schumacher et al. 2021). However, the timing of exercise intervention is still a matter of debate in the setting of prostatectomy. Researchers have examined the effect of specific interventions before or following surgery in prostate cancer patients (Singh et al. 2017b; Santa Mina et al. 2018; Blackwell et al. 2020; Baumann et al. 2022), with only two randomised controlled trials (Santa Mina et al. 2018; Blackwell et al. 2020) of structured exercise before prostatectomy. In these studies, home-based exercise (Santa Mina et al. 2018) and supervised high-intensity interval training (Blackwell et al. 2020) were feasible and led to improvements in physical function, psychological distress, cardiorespiratory fitness, and blood pressure, without any exercise-related adverse effects. In contrast, rehabilitation programmes following prostatectomy primarily focus on physiotherapy-led interventions particularly pelvic floor muscle training, although the efficacy of pelvic floor muscle training remains inconsistent (Baumann et al. 2022). Additionally, this intervention does not target the expected declines in body composition, physical capacity and QoL following surgery (Strassels et al. 2004).

Although we (Singh et al. 2017b) and others (Santa Mina et al. 2018; Blackwell et al. 2020) have demonstrated the potential of prehabilitative exercise in men with prostate cancer, it remains unclear whether commencing exercise before surgery, when patients are generally in better physical condition, would be superior to postoperative rehabilitation in terms of objective and patient-reported outcomes, hospital length of stay (LOS) and urinary incontinence. It may well be that initiating exercise before prostatectomy may mitigate surgery-related declines in physical function and QoL, considering that patients generally have better physical condition compared to the early postoperative period, as observed in a study conducted in patients with colorectal cancer (Gillis et al. 2014). As a result, we extend our previous work by asking, is it more efficacious to exercise the patient before surgery rather than rehabilitating the patient after prostatectomy?

## Materials and methods

**Fig. 1:**
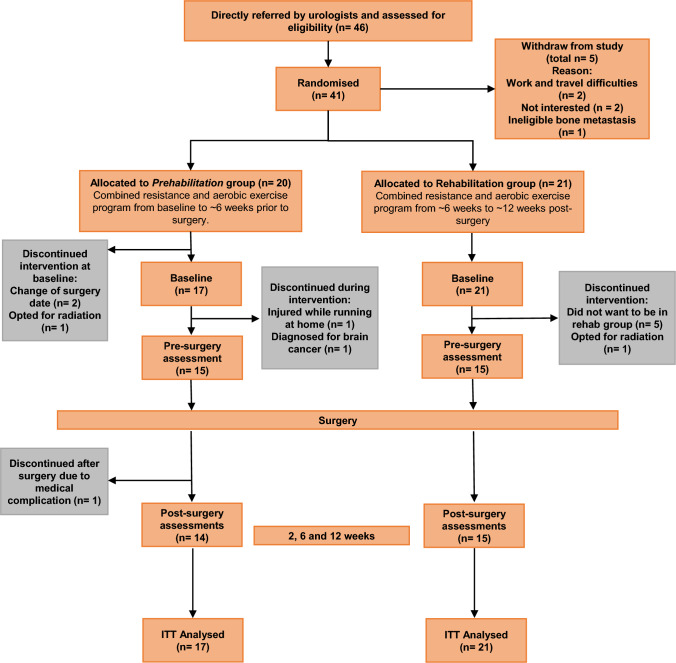
Study flow chart

Forty-six patients were referred for participation between June 2016 and September 2018 in Perth, Western Australia, and their progress through the study is shown in Fig. 1. Inclusion criteria included: histologically documented localised prostate cancer; minimum 7 weeks between baseline assessment and surgery for assessments and 6-weeks of exercise; and no acute illness, musculoskeletal, cardiovascular, or neurological disorder that would prevent patients from exercising as determined by their physician. Of these 46 patients, one was excluded due to bone metastasis diagnosis, two declined participation citing work/travel difficulties and two others were uninterested, resulting in 41 eligible patients. All patients obtained medical clearance and completed a health history questionnaire. Following randomisation (Prehabilitation, n = 20; Rehabilitation*,* n = 21), three prehabilitation participants withdrew before baseline assessments due to surgery date change (n = 2) and treatment change to radiation (n = 1), resulting in a study cohort of 38 patients. The study was approved by the Edith Cowan University Human Research Ethics Committee and the South Metropolitan Health Service, and all participants provided written informed consent.

### Study design

This randomised controlled trial compared the effectiveness of a 6-week supervised multimodal exercise programme before surgery (Prehabilitation) versus the identical exercise programme delivered post-surgery (Rehabilitation) in patients undergoing prostatectomy. Patients were randomly assigned using a computer random assignment programme to either group stratified for physical activity level (i.e., cut-off value of 150 min/week). Allocation was concealed using sealed opaque envelopes. Following familiarisation and baseline assessment, prehabilitation comprised combined resistance and aerobic exercise for 6 weeks pre-surgery with no formal intervention post-surgery, whilst rehabilitation exercise commenced 6 weeks post-surgery. All patients were instructed to rest and not exercise for at least 6 weeks after surgery to allow for recovery. Measurements were undertaken at baseline, pre-surgery, 6 and 12 weeks post-surgery. Quality of life and fatigue were additionally assessed at 2 weeks post-surgery, while urinary incontinence was assessed at 2, 6, and 12 weeks post-surgery. In this report, the time between baseline to pre-surgery is defined as the pre-surgery phase, the period from discharge to 6 weeks post-surgery as the early post-surgery phase, and between 6 and 12 weeks post-surgery as the late post-surgery phase (Figure S1).

### Exercise programme

Exercise was undertaken thrice weekly in small groups of 5–8 participants, supervised by accredited exercise physiologists in an exercise clinic. Each session was ~ 90 min and included resistance and aerobic exercises. Resistance training consists of exercises targeting major muscle groups, such as leg press, leg extension, leg curl, chest press, seated row, lat pulldown, triceps extension, biceps curl and calf raises (Singh et al. 2017a, 2017b, 2018). The intensity was set at 6–12 repetition maximum (RM) using 2–4 sets per exercise. Trunk-specific exercises, including plank, reverse bridge on a Swiss ball, and side planks, were also included, with progressively increasing training load (3 sets of 30–60 s) and decreasing rest time between sets.

The aerobic-based component involved various modes of exercise such as treadmill walking/jogging and cycling or rowing on a stationary ergometer with intensity set at 60–80% of the individual participant’s estimated maximum heart rate (220—age in years) for 20–30 min. All sessions commenced with a warm-up comprising low-level aerobic exercise (~ 60% maximum heart rate) and stretching exercises for the major muscle groups and concluded with a cooldown of stretching exercises. Rehabilitation participants were instructed to maintain their customary physical activity and dietary patterns before surgery.

### Primary and secondary endpoints

The primary study endpoint was dynamic muscle strength for the chest press and leg press using one-repetition maximum (1-RM) (Taaffe et al. 1999). Secondary endpoints were physical function, body composition, urinary incontinence, hospital LOS, fatigue, and QoL. Physical function was assessed using a battery of tests that included the repeated chair rise, stair climb, 400-m walk, and usual, fast and backward 6-m walk (Galvão et al. 2006). All tests were performed in triplicate except the 400-m walk which was performed once. Whole-body lean mass (LM), fat mass (FM), trunk fat mass, and body fat percentage were assessed by dual-energy x-ray absorptiometry (DXA, Hologic Discovery, Waltham, MA, USA). Quality of life was assessed using the global health domain, while fatigue was assessed using the fatigue subscale from the European Organization for Research and Treatment of Cancer Quality of Life Questionnaire-Core 30 version 3.0 (EORTC QLQ-C30) (Aaronson et al. 1993). Urinary incontinence was measured using the 24-h pad test calculated using the weight of urine loss (used—pre-weighed) at all time points post-surgery, and hospital LOS was assessed from medical records.

### Other measures

Demographic and clinical data including disease stage, comorbidities, surgical approach and medications were recorded and collected by self-report records. Height and body weight were assessed, with body mass index (BMI, kg.m^−2^) calculated. Physical activity was assessed by the Godin Leisure-Time Physical Activity Questionnaire (Godin and Shephard 1985). Prostate-specific antigen was measured commercially by an accredited Australian National Association of Testing Authorities (NATA) laboratory (Pathwest Diagnostics, Perth, WA, Australia).

### Statistical analysis

The sample size estimate was based on projected changes in muscle strength (Galvão et al. 2010). To achieve 95% power at an alpha level of 0.05 (two-tailed), 16 participants per group were required to detect a standardised mean difference in change of 0.5 (standard deviation of 0.4). (Faul et al. 2009). To account for an attrition rate of up to 30%, the goal was to recruit 21 patients per group.

Data were analysed using IBM SPSS version 27 (SPSS Inc., IBM Corp., Armonk, NY, USA). Normality of the distribution for outcome measures was evaluated using the Kolmogorov–Smirnov test. Differences in baseline characteristics between groups were assessed using independent t-tests or the Mann–Whitney U-test, as appropriate, for continuous data and chi-square for categorical data. Data were analysed on an intention-to-treat basis, with sensitivity analyses undertaken to ensure data robustness using complete cases approach (Thabane et al. 2013). Generalised estimating equations (GEE) were used to compare groups over time for the primary and secondary outcomes with follow-up tests undertaken if there was a significant group x time interaction or time effect. Results are presented as the mean and standard error (SE), median and interquartile range (IQR), and mean difference and 95% confidence interval (95% CI). All tests were two-tailed and an alpha level of 0.05 was required for statistical significance.

## Results

The 38 patients were aged 48 to 73 years, most were married (71.1%), without tertiary education (71.1%), and classed as moderately active or active (76.3%) based on the Godin Leisure-time Physical Activity Questionnaire (Table 1). BMI was 28.5 ± 3.4 kg.m^−2^ with most patients overweight or obese (86.9%). The median time since prostate cancer diagnosis was 1.0 month (IQR: 1.0 to 2.0 months), with median prostate-specific antigen (PSA) levels of 7.0 ng.ml^−1^ (IQR: 3.9 to 9.5 ng.ml^−1^) and Gleason Score of 7.0 (IQR: 7.0 to 7.0). Most patients underwent robotic-assisted laparoscopic prostatectomy (92.1%).

**Table 1:** Participant characteristics at baseline

Variables	Prehabilitation (N = 17)	Rehabilitation (N = 21)	p-value
Demographic			
Age, mean ± SD, yrs	62.1 ± 7.9	64.5 ± 5.7	0.283
Married, n (%)	13 (76.5%)	14 (66.7%)	0.508
Tertiary education, n (%)	5 (29.4%)	6 (28.6%)	0.955
Currently employed, n (%)	8 (47.1%)	11 (52.4%)	0.744
Current smoker, n (%) ^a^	2 (40.0%)	0 (0%)	0.020
Current drinker, n (%) ^a^	15 (93.8%)	20 (95.2%)	0.843
Godin leisure-time exercise, median (IQR)	21.0 (16.5 to 36.0)	20.0 (8.5 to 33.0)	0.281
Clinical			
BMI, mean ± SD, kg.m^−2^	27.9 ± 2.8	29.0 ± 3.8	0.321
BMI categories, n (%)			
Normal weight (BMI < 25 kg.m^−2^)	3 (17.6%)	2 (9.5%)	
Overweight (BMI ≥ 25 to < 30 kg.m^−2^)	10 (58.8%)	11 (52.4%)	0.556
Obese (BMI ≥ 30 kg.m^−2^)	4 (23.5%)	8 (38.1%)	
Time since diagnosis, median (IQR), mo ^a^	1.0 (0.5 to 2.0)	1.0 (1.0 to 2.0)	0.684
Number of medications, median (IQR) ^a^	2.0 (1.0 to 2.5)	1.0 (1.0 to 3.8)	0.619
Number of comorbidities, median (IQR) ^b^	2.0 (0.0 to 2.0)	1.0 (0.5 to 2.0)	0.999
PSA, median (IQR), ng.ml^−1 a^	6.7 (3.7 to 10.0)	6.7 (3.9 to 10.0)	0.602
Gleason score, median (IQR) ^a^	7.0 (7.0 to 7.0)	7.0 (7.0 to 7.0)	0.153
Gleason categories, n (%)			
Slow growing (Gleason ≤ 6)	0 (0.0%)	3 (18.8%)	
Fast-growing, moderately aggressive (Gleason = 7)	13 (86.7%)	11 (68.8%)	0.008
Fast-growing, aggressive (Gleason ≥ 8)	2 (13.3%)	2 (12.5%)	
Type of prostatectomy procedure			
Laparoscopic radical prostatectomy	15 (88.2%)	20 (95.2%)	0.371
Radical retropubic prostatectomy	2 (11.8%)	1 (4.8%)	

*BMI* body mass index, *IQR* interquartile range, *PSA* prostate-specific antigen, *SD* standard deviation

^a^Missing values: current smoker, n = 21; current drinker, n = 1; time since diagnosis, n = 2; number of medications, n = 9; PSA, n = 6; Gleason score, n = 7

^b^Cardiovascular disease, diabetes, hypertension, hypercholesterolemia, osteoporosis, depression

Three patients in prehabilitation and six from rehabilitation withdrew following baseline measurements preferring not to be in the rehabilitation group (n = 5), opting for radiation treatment (n = 1), diagnosis of brain cancer (n = 1), running injury not related to the exercise programme (n = 1), and medical complications after surgery (n = 1) (Fig. 1). Patients in prehabilitation attended 73.7% of scheduled exercise sessions while those in rehabilitation attended 93.7% of scheduled sessions. The prehabilitation group's lower attendance was attributed to patients attending medical appointments in relation to surgery. There were no exercise-related adverse events.

### Muscle strength

**Fig. 2:**
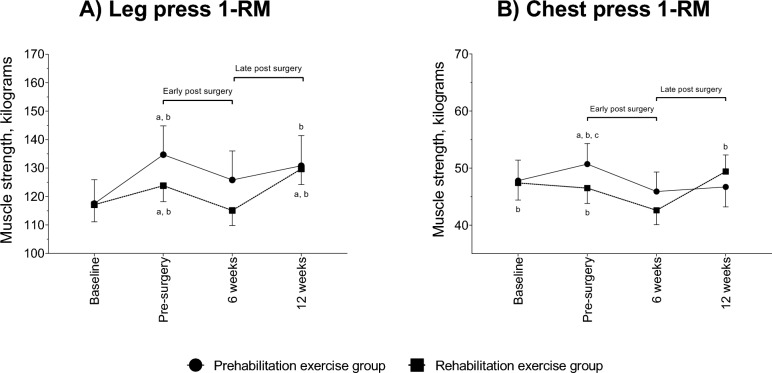
Muscle strength absolute values and change over the assessment time points. Results are presented as mean and standard error. ^a^statistically within-group change compared to baseline; ^b^statistically within-group change compared to 6-weeks post-surgery; ^c^statistically within-group change compared to 12-weeks post-surgery

There were no differences between groups at baseline (Fig. 2). Across the study time points, there was no significant interaction for leg or chest press strength; however, there was a significant effect of time (p < 0.001). In the pre-surgery phase, prehabilitation significantly improved both leg (17.2 kg, p < 0.001) and chest press strength (2.9 kg, p = 0.001) with rehabilitation also experiencing a significant increase of 6.7 kg for leg press (p < 0.001). However, both groups exhibited significant reductions for chest (Prehabilitation: 4.8 kg, p < 0.001; Rehabilitation: 3.9 kg, p < 0.001) and leg press (Prehabilitation: 8.9 kg, p = 0.024; Rehabilitation: 8.7 kg, p = 0.012) strength in the early post-surgery phase. During late post-surgery phase, significant improvements of 5.0 kg (p = 0.003) and 14.6 kg (p < 0.001) were observed in leg press for both prehabilitation and rehabilitation*,* respectively, with only rehabilitation improving chest press strength by 6.8 kg (p < 0.001). As a result, when comparing 12 weeks post-surgery to baseline, there was no significant difference in muscle strength between prehabilitation and rehabilitation. Comparable results were observed when analysing complete cases except for leg press strength (p = 0.042) which was higher in prehabilitation at 12 weeks post-surgery compared to baseline (Table S1).

### Physical function

**Fig. 3:**
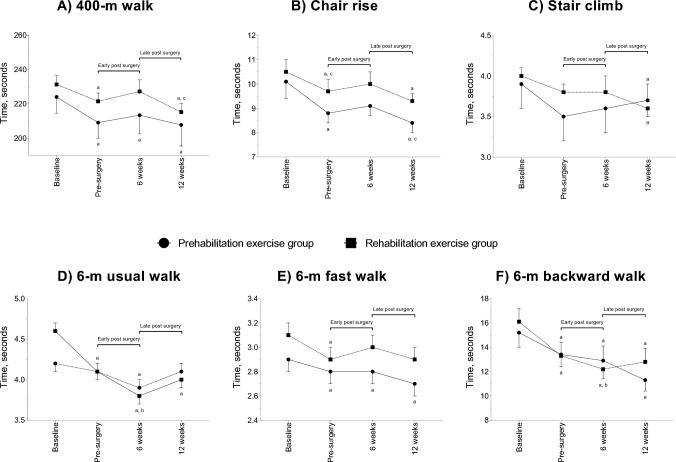
Physical function absolute values and change over the different assessment time points. Results are presented as mean and standard error. ^a^statistically within-group change compared to baseline; ^b^statistically within-group change compared to pre-surgery; ^c^statistically within-group change compared to 6-weeks post-surgery

There was no difference between groups at baseline for physical function (p = 0.060–0.685). Over the study period, there were no significant interactions except for 6-m usual walk (p = 0.033) and a significant time effect for 400-m walk, chair rise, stair climb, 6-m fast and backward walk tests (p =  < 0.001–0.001) (Fig. 3). During the pre-surgery phase, there were significant reductions (improvement) for prehabilitation of –  14.9 s in the 400-m walk, –  1.3 s in chair rise, –  0.2 s and –  1.8 s in 6-m fast and backward walk tests, respectively, (p =  < 0.001–0.028). The rehabilitation group also had significant reductions of –  9.8 s in the 400-m walk, –  0.8 s in chair rise, –  0.4, –  0.2 and –  2.8 s for 6-m usual, fast, and backward walk tests, respectively, (p =  ≤ 0.001–0.012). Physical function during the early post-surgery phase (p = 0.152–1.000) was maintained through to the late post-surgery phase (p = 0.170–1.000) for prehabilitation except for a 0.7 s reduction in the chair rise test (p = 0.004). During the early post-surgery phase, rehabilitation improved with a reduced 6-m usual (–  0.3 s, p = 0.019) and backward walk time (–  1.1 s, p = 0.028), however, chair rise time increased (0.3 s, p = 0.033). The 400-m walk (–  12.0 s, p = 0.005) was the only improvement in rehabilitation during late post-surgery phase.

When comparing 12 weeks post-surgery to baseline, prehabilitation had significant improvement of –  16.3 s (95% CI: –  26.2 to –  6.3 s, p < 0.001) in 400-m walk, –  1.7 s (95% CI: –  3.0 to –  0.4 s, p = 0.004) in chair rise, –  0.3 s (95% CI: –  0.5 to –  0.0 s, p = 0.050) in stair climb, − 0.2 s (95% CI: –  0.4 to –  0.0 s, p = 0.047) in 6-m fast walk and –  3.9 s (95% CI: –  6.6 to –  1.3 s, p = 0.001) in backwards walk. In the rehabilitation group, there was a significant improvement of –  16.1 s (95% CI: –  24.8 to –  7.4 s, p < 0.001) in 400-m walk, –  1.2 s (95% CI: –  2.3 to –  0.2 s, p = 0.010) in chair rise, –  0.4 s (95% CI: –  0.7 to –  0.0 s, p = 0.034) in stair climb, –  0.5 s (95% CI: –  0.9 to –  0.2 s, p < 0.001) in 6-m usual walk and –  3.3 s (95% CI: –  5.6 to –  1.0 s, p = 0.001) in backwards walk following exercise post-surgery compared to baseline. As a result, there was no significant difference in physical function between groups at 12 weeks post-surgery. Similar results were observed when analysing complete cases except for a significant reduction (improvement) in chair rise (p = 0.036) for rehabilitation comparing 6 to 12 weeks post-surgery (Table S2).

### Body composition

At baseline, prehabilitation had lower whole-body LM (p = 0.007) and trunk FM (p = 0.038) compared to rehabilitation (Table 2). There were no significant interactions for body composition (p = 0.080–0.586) except for body fat % (p = 0.029), with a significant effect of time for LM, FM, and trunk FM (p =  < 0.001–0.041). No differences were found in either group during the pre-surgery phase; however, in early post-surgery phase, both groups lost LM (Prehabilitation 1.6 kg, p = 0.008; Rehabilitation 1.1 kg, p = 0.004) with prehabilitation having an increase of 1.7% in percent body fat (p = 0.007). There were no differences in body composition during the late post-surgery phase. When comparing 12 weeks post-surgery to baseline, there was a significant decrease in whole-body FM of 1.1 kg (95% CI: –  2.1 to –  0.2 kg, p = 0.008) and trunk FM of 0.7 kg (95% CI: –  1.3 to –  0.2 kg, p = 0.005) in rehabilitation with no significant differences for prehabilitation. When analysing complete cases, comparable results were observed for both groups (Table S3).

**Table 2:** Body composition outcomes at baseline, pre-surgery, and 6 and 12 weeks post-surgery

Outcome variables	Baseline	Pre-surgery	6-weeks	12-weeks	p-value		Comparison between assessments*
	Mean ± SE	Mean ± SE	Mean ± SE	Mean ± SE	Time	Group × time	
Whole-body fat mass, kg							
Prehabilitation	24.8 ± 1.3	23.5 ± 1.3	24.6 ± 1.2	24.1 ± 1.2	0.041	0.080	–
Rehabilitation	28.6 ± 1.5	28.2 ± 1.4	27.8 ± 1.4	27.4 ± 1.4			a > c, d
Whole-body lean mass, kg							
Prehabilitation	54.7 ± 1.0	54.7 ± 0.8	53.1 ± 1.1	53.8 ± 1.0	< 0.001	0.586	a, b > c
Rehabilitation	60.8 ± 1.8	60.7 ± 1.9	59.6 ± 1.9	60.6 ± 2.0			a, b > c
Trunk fat mass, kg							
Prehabilitation	12.7 ± 0.7	11.8 ± 0.7	12.3 ± 0.6	12.1 ± 0.7	0.030	0.174	–
Rehabilitation	15.3 ± 0.9	15.0 ± 0.8	14.7 ± 0.8	14.6 ± 0.8			a > c, d
Body fat percentage, %							
Prehabilitation	29.9 ± 1.0	28.7 ± 1.0	30.4 ± 0.9	29.7 ± 0.9	0.001	0.029	b < c
Rehabilitation	30.6 ± 0.7	30.4 ± 0.6	30.4 ± 0.6	29.8 ± 0.7			–

*Within-group multiple comparisons for baseline, pre-surgery, 6 and 12-weeks post-surgery, with a Bonferroni-corrected p < 0.05; (a) Baseline, (b) Pre-surgery, (c) 6-weeks post-surgery, (d) 12-weeks post-surgery

### Quality of life and fatigue

At baseline, there was no difference between groups for QoL and fatigue (Table 3). Over the course of the study, there were no interactions but a significant time effect for both QoL and fatigue (p < 0.001). During the pre-surgery phase, fatigue was significantly reduced (p = 0.002) for prehabilitation with no change in rehabilitation. Both groups had an increase in fatigue at 2 weeks post-surgery which was then reduced at 6 and 12 weeks post-surgery. There was no change in QoL pre-surgery; however, there was a substantial decline in both groups at 2 weeks post-surgery which then recovered to baseline levels at 12 weeks post-surgery. Similar results were observed for both groups in complete case analyses (Table S4).

**Table 3:** Urinary incontinence, quality of life and fatigue at all assessment time points

Outcome variables	Baseline	Pre-surgery	2-weeks	6-weeks	12-weeks	p-value		Comparison between assessments*
	Mean ± SE	Mean ± SE	Mean ± SE	Mean ± SE	Mean ± SE	Time	Group × time	
Urinary incontinence, g								
Prehabilitation	–	–	345.1 ± 113.7	124.8 ± 48.8	82.4 ± 47.2	< 0.001	0.884	–
Rehabilitation	–	–	410.2 ± 148.7	104.9 ± 30.1	71.7 ± 42.7			c > e
Quality of life^#^								
Prehabilitation	84.8 ± 3.8	83.3 ± 3.2	63.8 ± 5.2	74.7 ± 3.5	81.4 ± 4.3	< 0.001	0.836	a, b, d, e > c
Rehabilitation	82.9 ± 3.3	79.1 ± 4.9	60.0 ± 4.5	75.0 ± 2.6	81.5 ± 2.8			a, b, d, e > c; a > d
Fatigue								
Prehabilitation	13.1 ± 3.5	6.6 ± 2.1	47.9 ± 7.8	28.5 ± 6.2	19.4 ± 4.0	< 0.001	0.121	b < e, a < d < c
Rehabilitation	10.5 ± 3.0	12.5 ± 4.1	31.9 ± 3.2	14.3 ± 4.1	11.2 ± 4.7			a, b, d, e < c

^#^Global health domain;

*Within-group multiple comparisons for baseline, pre-surgery, 2, 6 and 12-weeks post-surgery, with a Bonferroni-corrected p < 0.05; (a) Baseline, (b) Pre-surgery, (c) 2-weeks post-surgery, (d) 6-weeks post-surgery, (e) 12-weeks post-surgery

### Urinary incontinence and length of hospital stay

At 2 weeks post-surgery, there was no difference between groups for urinary incontinence (p = 0.790) (Table 3). Across the post-surgery time points, there was no interaction (p = 0.884) but a significant time effect (p < 0.001). Rehabilitation had a significant reduction of 338.5 g (95% CI: –  615.6 to –  61.4 g) in the 24-h pad test (p = 0.010) between 2 and 12 weeks post-surgery, while prehabilitation reduction approached statistical significance (262.7 g, p = 0.067). Results were similar in sensitivity analyses (Table S4). For hospital LOS, there was no difference between groups (Prehabilitation, 2.9 ± 1.4 days vs. Rehabilitation, 2.5 ± 1.3 days; p = 0.473).

## Discussion

In this study, we compared prehabilitation and rehabilitation supervised exercise in the setting of prostatectomy. There were three main findings. First, 6 weeks of supervised exercise before prostatectomy enhanced muscle strength, physical function at pre-surgery and at 6 weeks post-surgery despite reductions in lean mass so that even though these patients were in the postsurgical recovery period, their physical performance was comparable to or better than prior to surgery when compared to rehabilitation. In addition, improvements in strength and function were accompanied by reduced fatigue prior to surgery. Second, for those initiating exercise post-surgery, supervised exercise helped recoup losses and enhance muscle strength and physical function. Third, both groups experienced comparable resolution in urinary incontinence with similar hospital LOS.

We demonstrated that commencing exercise 6 weeks before surgery substantially improved muscle strength and physical function, which may act to buffer the effects of surgery. As a result of the patient’s improved reserve capacity, declines that do occur in strength and function do not typically result in the patient falling below their pre-exercise levels. In addition, we observed a reduction in fatigue levels in those who exercised before surgery. These findings support previous research in the prehabilitation setting (Singh et al. 2017b; Santa Mina et al. 2018; Blackwell et al. 2020) and highlight the importance of implementing exercise therapy early after a prostate cancer diagnosis to minimise musculoskeletal effects from prostatectomy (Strassels et al. 2004). Exercising at an early stage before surgery provides an opportune time to intervene and takes advantage of surgical wait times when patients are in a better condition to exercise compared to the early post-operative period (Santa Mina et al. 2015).

The rehabilitation programme also showed important benefits for patients who are unable to engage in exercise before surgery due to numerous medical appointments and commitments or the short time between diagnosis and prostatectomy (e.g., the urgency of the prostate cancer condition and surgeon availability). Exercise that commenced after this initial period, with rehabilitative intent, is also beneficial to recoup losses and enhance muscle strength and physical function while maintaining or improving QoL. These findings are clinically important as increasing physical reserve capacity is associated with reduced risk of postoperative complications and all-cause mortality in cancer patients (Ezzatvar et al. 2021).

Although improvements in both muscle strength and physical function resulted from the training program in both groups, there was an absence of improvement in whole body LM. Consequently, improvements in strength and function are likely attributable to neural adaptations to exercise (Carli and Zavorsky 2005). From pre-surgery to 6 weeks post-surgery, the decrease in whole-body LM for both groups could be attributed to bed rest, physical inactivity and possibly dietary changes (Dirks et al. 2016), although these were not tracked in our study. Despite having a structured and supervised exercise program, a 6-week program may be insufficient to induce substantial increases in lean mass and reductions in fat mass (Singh et al. 2017b). Nevertheless, a combined resistance and aerobic exercise programme is still a positive step towards incorporating non-surgical and non-pharmaceutical therapies within a patient treatment plan to improve long-term health outcomes and prevent adverse effects (Bodai et al. 2018). A comprehensive prehabilitative approach, including nutrition and psychological support interventions (Molenaar et al. 2019), should be considered to prevent chronic inflammation (Pedersen and Febbraio 2012), reduce cardiovascular disease risk (Chen et al. 2019), and alleviate cancer-related fatigue (Newton et al. 2018) throughout the prostate cancer care continuum. In our study, pre-surgery exercise was associated with a reduction in fatigue of 6.5 points, meeting the MID reported for the EORTC QLQ-C30 (Nordin et al. 2016). This result is clinically important as it provides guidelines for pre-surgical exercise prescriptions to ameliorate cancer-related fatigue. As expected, fatigue increased in the immediate post-surgical period in both groups but gradually resolved towards baseline levels at 6- and 12-weeks post-surgery. Commencing exercise early and maintaining exercise for extended periods, even after prostatectomy, may be crucial to preserve or enhance body composition (Lopez et al. 2022) and improve fatigue.

The evidence regarding prehabilitative exercise for reducing post-operative urinary incontinence is conflicting in patients with prostate cancer (Xiangyun et al. 2022). In a meta-analysis that included 14 studies, the researchers concluded that preoperative pelvic floor muscle training did not significantly reduce urinary incontinence rate at 1, 3, 6 and 12 months after surgery (Xiangyun et al. 2022). Additionally, evidence was inconclusive for studies undertaken in patients undergoing robotic-assisted laparoscopic radical prostatectomy (Xiangyun et al. 2022), which was the predominant surgical technique in our study. Indeed, our findings are that prehabilitative exercise, including resistance, aerobic and trunk-specific exercises, did not effectively reduce post-surgery urinary incontinence. This may be explained by the quality, effectiveness, and less invasive approach of robotic-assisted laparoscopic radical prostatectomy, including less blood loss and pain, and faster recovery. Moreover, this surgical approach is already associated with shorter hospital stays, which may explain the lack of difference between groups on hospital LOS after prostatectomy.

The strengths of this study include a comprehensive clinic-based exercise programme with supervision, a high attendance rate, a battery of physical function and muscle strength assessments, and use of DXA for body composition. Nevertheless, there are limitations worthy of comment. Most participants underwent a less invasive surgical technique, which may have limited our ability to observe the effects of exercise on urinary incontinence and hospital LOS. Additionally, the study participants may not represent all prostate cancer patients undergoing surgery, as they agreed to participate in an exercise intervention during a challenging period. Lastly, the study did not have a long-term follow-up to assess the persistence of exercise over time, such as 6 months or longer. Nevertheless, we found that a relatively brief programme of supervised resistance and aerobic exercise undertaken either before or after prostatectomy can be safely undertaken and results in improvements in muscle strength and physical function. The time period between diagnosis and surgery, despite being a relatively short time frame, provides a window of opportunity to introduce targeted exercise programmes.

In conclusion, exercise before prostatectomy enhances muscle strength and physical function, and reduces fatigue in patients with prostate cancer. If exercise before surgery is not possible, starting supervised exercise after prostatectomy may recoup losses and enhance muscle strength, physical function and potentially body composition while maintaining QoL. It may well be that exercising both before and after surgery may provide the most significant physical enhancement. Clinicians should encourage patients to engage in exercise either before or as soon as possible after the acute post-operative phase to assist with their recovery.

## Supplementary Information

Below is the link to the electronic supplementary material.

Supplementary file1 (PDF 93 KB)

## Data Availability

The datasets generated during and/or analysed during the current study are available from the corresponding author upon reasonable request.
