# Supplementary material for: Prehabilitative versus rehabilitative exercise in prostate cancer patients undergoing prostatectomy
Source: J Cancer Res Clin Oncol. 2023 Sep 15;149(18):16563–73. doi: 10.1007/s00432-023-05409-3 (PMC10645629; doi:10.1007/s00432-023-05409-3)

Table S1. Sensitive analysis for muscle strength outcomes at baseline, pre-surgery, 6 and 12 weeks post-surgery.

Outcome Variables	Baseline<br>Mean ± SE	Pre-surgery<br>Mean ± SE	6-weeks<br>Mean ± SE	12-weeks<br>Mean ± SE	p-value<br>Time	 Group x Time	Comparison between assessments*
**Leg press, kg**							
Prehabilitation	116.7 ± 10.1	136.0 ± 11.7	126.0 ± 11.9	131.0 ± 12.1	< 0.001	0.796	a, c < b, d
Rehabilitation	120.1 ± 7.5	126.6 ± 7.1	117.7 ± 6.5	131.8 ± 6.9			a, c < b, d
**Chest press, kg**							
Prehabilitation	48.0 ± 4.4	51.0 ± 4.3	46.1 ± 4.1	46.9 ± 4.2	< 0.001	0.946	a, c, d < b
Rehabilitation	49.6 ± 4.0	48.1 ± 3.7	44.5 ± 3.5	51.4 ± 3.8			a, b, d > c; b < d

*Within-group multiple comparisons for baseline, pre-surgery, 6 and 12-weeks post-surgery, with a Bonferroni-corrected $P < 0.05$, (a) Baseline, (b) Pre-surgery, (c) 6-weeks post-surgery, (d) 12-weeks post-surgery.

Table S2. Sensitive analysis for physical function outcomes at baseline, pre-surgery, 6 and 12 weeks post-surgery.

Outcome Variables	Baseline Mean ± SE	Pre-surgery Mean ± SE	6-weeks Mean ± SE	12-weeks Mean ± SE	p-value Time	p-value Group x Time	Comparison between assessments*
**400-m walk, sec**							
Prehabilitation	223.0 ± 11.2	206.4 ± 10.7	211.5 ± 12.6	205.9 ± 14.1	< 0.001	0.325	a > b, c, d
Rehabilitation	233.7 ± 6.7	223.2 ± 6.4	229.3 ± 8.2	217.3± 6.3			a > b, d; c > d
**Chair Rise, sec**							
Prehabilitation	10.1 ± 0.9	8.7 ± 0.5	9.1 ± 0.5	8.6 ± 0.5			a > b, d; c > d
Rehabilitation	10.4 ± 0.7	9.7 ± 0.6	10.0 ± 0.6	9.2 ± 0.4	< 0.001	0.277	a > b, d; c > d
**Stair Climb, sec**							
Prehabilitation	4.0 ± 0.3	3.8 ± 0.3	3.8 ± 0.3	3.8 ± 0.3	0.002	0.699	-
Rehabilitation	3.9 ± 0.2	3.8 ± 0.2	3.8 ± 0.2	3.6 ± 0.1			a > d
**6-m usual walk, sec**							
Prehabilitation	4.2 ± 0.2	4.1 ± 0.1	3.9 ± 0.1	4.1 ± 0.1	< 0.001	0.012	a > c
Rehabilitation	4.6 ± 0.2	4.2 ± 0.1	3.9 ± 0.1	4.1 ± 0.1			a > b, c, d; b > c
**6-m fast walk, sec**							
Prehabilitation	3.0 ± 0.1	2.8 ± 0.1	2.8 ± 0.1	2.8 ± 0.1	< 0.001	0.590	a > b, c, d
Rehabilitation	3.1 ± 0.1	2.8 ± 0.1	3.0 ± 0.1	2.9 ± 0.1			a > b
**6-m backward walk, sec**							
Prehabilitation	15.6 ± 1.3	13.6 ± 1.1	13.2 ± 1.3	12.0 ± 0.9	< 0.001	0.372	a > b, c, d
Rehabilitation	15.6 ± 1.2	13.1 ± 0.9	11.8 ± 0.8	12.0 ± 0.9			a > b, c, d; b > c

*, Within-group multiple comparisons for baseline, pre-surgery, 6 and 12-weeks post-surgery, with a Bonferroni-corrected P < 0.05, (a) Baseline, (b) Pre-surgery, (c) 6-weeks post-surgery, (d) 12-weeks post-surgery.

Table S3. Sensitive analysis for body composition outcomes at baseline, pre-surgery, 6 and 12 weeks post-surgery.

Outcome Variables	Baseline	Pre-surgery	6-weeks	12-weeks	p-value		Comparison between assessments*
	Mean ± SE	Mean ± SE	Mean ± SE	Mean ± SE	Time	Group x Time	
**Whole-body fat mass, kg**							
Prehabilitation	24.8 ± 1.3	23.3 ± 1.3	24.5 ± 1.1	24.0 ± 1.1	0.051	0.077	-
Rehabilitation	26.4 ± 1.6	26.1 ± 1.5	25.7 ± 1.6	25.3 ± 1.5			-
**Whole-body Lean mass, kg**							
Prehabilitation	55.3 ± 0.9	55.1 ± 0.8	53.6 ± 1.1	54.3 ± 1.0	< 0.001	0.673	a, b > c
Rehabilitation	58.8 ± 2.2	58.7 ± 2.2	57.6 ± 2.2	58.6 ± 2.4			a, b > c
**Trunk fat mass, kg**							
Prehabilitation	13.2 ± 0.8	12.2 ± 0.8	12.8 ± 0.7	12.5 ± 0.7	0.040	0.175	-
Rehabilitation	14.2 ± 1.0	13.9 ± 0.9	13.6 ± 0.9	13.4 ± 0.9			a > c, d
**Body fat percentage, %**							
Prehabilitation	29.6 ± 1.0	28.5 ± 1.1	30.2 ± 0.9	29.4 ± 0.9	0.003	0.040	b < c
Rehabilitation	29.8 ± 0.8	29.6 ± 0.7	29.6 ± 0.7	29.0 ± 0.7			-

*, Within-group multiple comparisons for baseline, pre-surgery, 6 and 12-weeks post-surgery, with a Bonferroni-corrected P < 0.05, (a) Baseline, (b) Pre-surgery, (c) 6-weeks post-surgery, (d) 12-weeks post-surgery.

Table S4. Sensitive analysis for urinary incontinence, quality of life and fatigue at all assessment time points.

Outcome Variables	Baseline	Pre-surgery	2-weeks	6-weeks	12-weeks	p-value		Comparison between assessments*
	Mean ± SE	Mean ± SE	Mean ± SE	Mean ± SE	Mean ± SE	Time	Group x Time	
**Urinary Incontinence, g**								
Prehabilitation	-	-	345.1 ± 113.7	124.8 ± 48.8	82.4 ± 47.2	< 0.001	0.884	-
Rehabilitation	-	-	410.2 ± 148.7	104.9 ± 30.1	71.7 ± 42.7			c > e
**Quality of life** #								
Prehabilitation	84.6 ± 4.4	82.0 ± 3.6	64.8 ± 5.5	75.0 ± 3.6	81.4 ± 4.6	< 0.001	0.886	a, b, e > c
Rehabilitation	83.3 ± 4.4	77.3 ± 5.3	60.6 ± 5.8	74.3 ± 3.3	80.3 ± 3.3			a, d, e > c
**Fatigue**								
Prehabilitation	12.0 ± 3.5	6.0 ± 2.3	44.5 ± 8.3	24.8 ± 5.9	18.8 ± 4.4	< 0.001	0.185	a, b, d, e < c; b < a, d
Rehabilitation	12.1 ± 3.9	12.1 ± 4.4	30.3 ± 3.5	13.1 ± 4.5	9.1 ± 3.1			a, b, d, e < c

Global health domain. *Within-group multiple comparisons for baseline, pre-surgery, 2, 6 and 12-weeks post-surgery, with a Bonferroni-corrected $p < 0.05$, (a) Baseline, (b) Pre-surgery, (c) 2-weeks post-surgery, (d) 6-weeks post-surgery, (e) 12-weeks post-surgery.

Figure S1. Study design

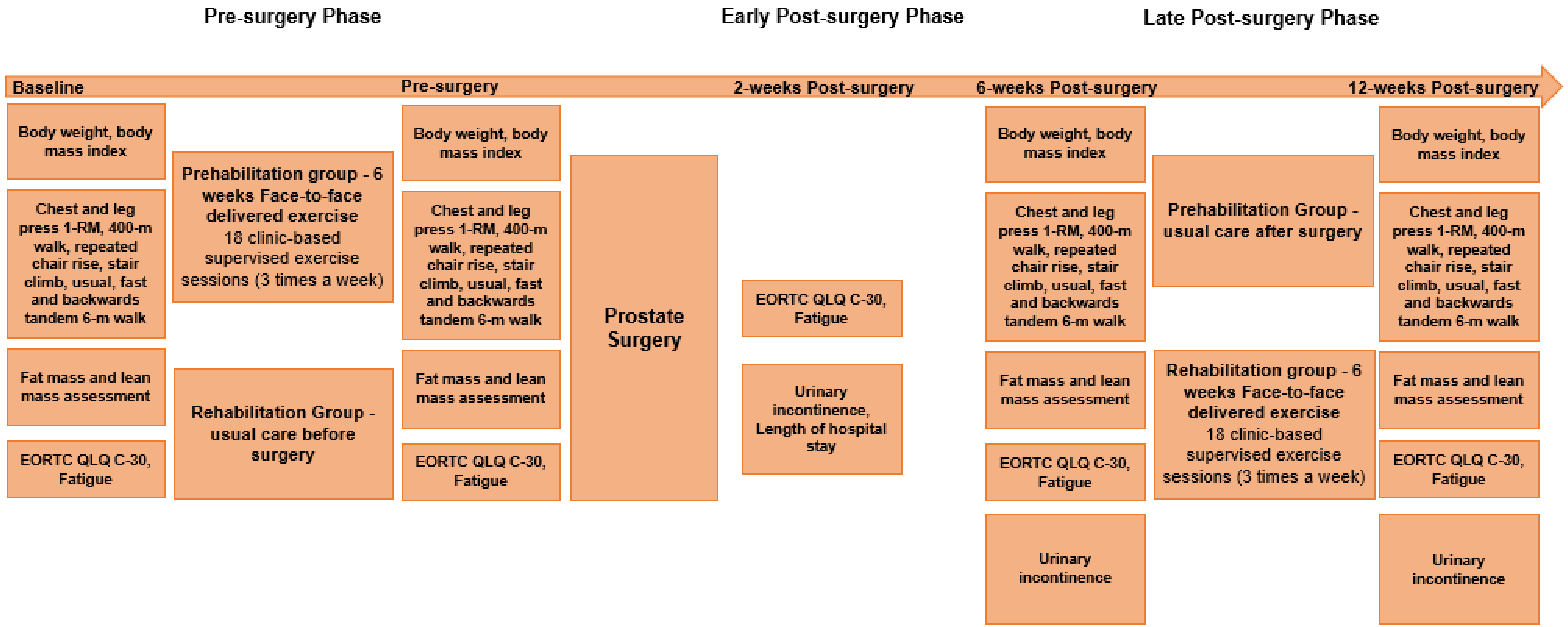

Supplement: Supplementary file 1 — Supplementary file1 (PDF 93 KB) [file 432_2023_5409_MOESM1_ESM.pdf]
